# Preparation and Biocompatible Surface Modification of Redox Altered Cerium Oxide Nanoparticle Promising for Nanobiology and Medicine

**DOI:** 10.3390/bioengineering3040028

**Published:** 2016-11-03

**Authors:** Himansu Sekhar Nanda

**Affiliations:** 1 Physical Sciences and Engineering Division, King Abdullah University of Science and Technology, Thuwal 23955-6900, Saudi Arabia; hnanda@ntu.edu.sg or binodinitifr@gmail.com; 2 School of Materials Science and Engineering, Nanyang Technological University, Singapore 639798, Singapore

**Keywords:** cerium oxide nanoparticle, biocompatible, surface modification, aqueous dispersible, doped and co-doped, nanobiology and medicine

## Abstract

The biocompatible surface modification of metal oxide nanoparticles via surface functionalization technique has been used as an important tool in nanotechnology and medicine. In this report, we have prepared aqueous dispersible, trivalent metal ion (samarium)-doped cerium oxide nanoparticles (SmCNPs) as model redox altered CNPs of biological relevance. SmCNP surface modified with hydrophilic biocompatible (6-{2-[2-(2-methoxy-ethoxy)-ethoxy]-ethoxy}-hexyl) triethoxysilane (MEEETES) were prepared using ammonia-induced ethylene glycol-assisted precipitation method and were characterized using a variety of complementary characterization techniques. The chemical interaction of functional moieties with the surface of doped nanoparticle was studied using powerful ^13^C cross polarization magic angle sample spinning nuclear magnetic resonance spectroscopy. The results demonstrated the production of the extremely small size MEEETES surface modified doped nanoparticles with significant reduction in aggregation compared to their unmodified state. Moreover, the functional moieties had strong chemical interaction with the surface of the doped nanoparticles. The biocompatible surface modification using MEEETES should also be extended to several other transition metal ion doped and co-doped CNPs for the production of aqueous dispersible redox altered CNPs that are promising for nanobiology and medicine.

## 1. Introduction

Nanotechnology has attracted several nanomaterials for their potential applications in biology and medicine [1]. Cerium oxide nanoparticles (CNPs) have been investigated for their potential promise in treatment of several oxidative stress related disorders including cancer [2,3,4,5,6]. Due to the co-existence and auto-regeneration of Ce valence states (Ce^3+^ and Ce^4+^), these nanoparticles can act as both a pro-oxidant and an antioxidant [4,5]. Reactive oxygen and nitrogen species (ROS and RNS) are formed as a byproduct of the oxygen and nitrogen metabolism in our body [5]. The basal level of ROS and RNS are necessary for cell signaling while the excess are detrimental to cells and tissues. Therefore, the excess of these reactive species are constantly monitored by the natural antioxidant enzymes present in our body [5]. The unusual rise in the level of ROS and RNS during oxidative stress conditions such as atherosclerosis, cancer, diabetics, rheumatoid arthritis, cardiovascular diseases, chronic inflammation, and aging demands antioxidant therapy [7]. CNPs have been well investigated as an antioxidant nanomedicine to scavenge these reactive species and have shown to reduce the oxidative stress in cell and tissue sites [2,3,4,5]. Moreover, CNPs have also been investigated as a strong oxidant and they inhibit the cancer progression toxic to tumor cells and nontoxic to stromal cells [2,4,6,8].

Apart from the significant biological importance of pure CNPs, doping, and co-doping of CNPs with other transition metal ions has been of great interest in the development of redox altered CNPs for various nanobiological applications. These redox altered CNPs have demonstrated enhanced catalytic, optical, magnetic, and luminescent properties and have been investigated for their applications in imaging and therapy [9,10,11,12,13]. For example, the optical emission of CNPs could be enhanced with trivalent europium doping and the Eu-doped CNPs are promising in bioimaging applications [9,10,11]. CNPs co-doped with a sensitizer dopant ion such as ytterbium and one of the emitter dopant ions—such as erbium, holmium, thulium, and praseodymium—has been used for production of upconversion CNPs for their potential application in imaging and therapuetics [12,13]. Samarium doped CNPs were synthesized to blunt the antioxidant effects and has been used for demonstrating the role of redox switch (Ce^3+^/Ce^4+^) in antioxidant properties of CNP [14,15,16]. Gadolinium doped CNPs were synthesized for the nanotoxicity studies [15]. Transition metal ions such as Mn, Fe, Co, and Ni have been doped into CNPs to alter room temperature ferromagnetism and incorporate the magnetic properties in CNPs [17]. Overall, dopants were used for production of redox altered CNPs by altering the trivalent state of Ce in Ce^3+^/Ce^4+^ redox switch, keeping the oxygen vacancies almost the same [14]. All these studies demonstrated a progressive importance of doped and co-doped CNPs in various nanobiological applications.

Therefore, there is a burgeoning need to develop an appropriate synthetic procedure for production of aqueous dispersible doped/co-doped CNPs of biological relevance. Biocompatible surface modification using chemical (citrate, cyclodextrin, polymers, poly ethylene glycol (PEG), organosilanes etc.) and biological (Heparin) functional moieties has been used as a fundamental tool to reduce the nanoparticle agglomeration and aggregation for possible nanobiological applications [18,19,20,21]. Biocompatible surface modification of the nanoparticles could generate a biomimetic surface for reduction of nonspecific binding at biological milieu [18]. The stated aim of the study was to apply the similar concept of surface modification to a trivalent metal ion (samarium)-doped CNP (SmCNP) as a model for production of aqueous dispersible redox altered CNPs of biological relevance. The surfaces of SmCNPs were modified using hydrophilic biocompatible (6-{2-[2-(2-methoxy-ethoxy)-ethoxy]-ethoxy}-hexyl) triethoxysilane (MEEETES) as a functional chemical moiety. MEEETES surface modified SmCNPs were produced via ammonia-induced ethylene glycol-assisted precipitation method. The surface modification was carried out in situ in a single and two step synthetic procedure. The surface modified doped nanoparticles were studied through a series of physicochemical characterizations.

## 2. Experimental

### 2.1. Preparation and Surface Modification of Samarium Doped Cerium Oxide Nanoparticles

Samarium doped cerium oxide nanoparticles (SmCNPs) were prepared by ammonia (NH_3_)-induced ethylene glycol (EG)-assisted precipitation method. The preparation protocol was designed to introduce 20 mol % of Sm (III) as a dopant for production of SmCNPs. A combination of salts of Ce and Sm (Ce(NO_3_)_3_·6H_2_O and Sm(NO_3_)_3_·6H_2_O) in a 4:1 molar ratio was used as precursor materials for the preparation of SmCNPs.

Briefly, 7.8 mL (0.12 mol) of EG (99%, Sigma Aldrich, St. Louis, MO, USA) was added to 92.2 mL of deionized water (Milli-Q^®^ Direct Water Purification System, Merck Millipore, Billerica, MA, USA) in a 250 mL two neck round bottom distilling flask at 50 °C. The reaction was carried out in a silicone bath reflux condenser system under constant magnetic stirring. 4.13 g of Ce(NO_3_)_3_·6H_2_O (0.01 mol) and 1.06 g of Sm(NO_3_)_3_·6H_2_O (0.002 mol) as precursor salts were added into the solution of EG and water. After complete dissolution of precursor salts, 5 mL of ammonium hydroxide (NH_4_OH) (29.44%, Fisher Scientific, Pittsburgh, PA, USA) was added until the pH of the reaction mixture became 9.6. The solution was kept under constant magnetic stirring at 750 rpm and was allowed to react until the reaction mixture turned into yellow (as an indication of the formation of SmCNPs). After the confirmation of formation of SmCNPs, the stirring was stopped and the entire volume was subjected to vacuum filtration using standard Whatman (Ø = 110 mm and grade 589/3) filter paper over a Buchner funnel. The filtered nanomaterials were subjected to alternate wash with ethanol and deionized water (six times each) and dried overnight in a fume hood. The dried nanomaterials were crushed and ground in a ceramic mortar using a pestle and suspended in deionized water using sonication. The suspended nanoparticles were freeze-dried using a freeze-drier (Labconco Corporation, Kansas City, MO, USA). The freeze-dried nanoparticles were crushed and ground using the ceramic mortar and pestle to ensure the production of fine and powdered SmCNPs.

In order to create biocompatible surface in produced SmCNPs, in situ surface modification of nanoparticles was carried out as the part of the above synthetic procedure. A similar preparation scheme was followed up to the formation of SmCNPs. The surface modification was followed as an immediate step of the above synthetic procedure. Instead of nanoparticle (SmCNPs) recovery, 400 µL (20 mM) of (6-{2-[2-(2-Methoxy-ethoxy)-ethoxy]-ethoxy}-hexyl)triethoxysilane (MEEETES) (SiKEMIA) was added to entire 100 mL of reaction volume under an inert atmospheric condition and was allowed to react overnight until the solution turned to milky yellow (an indication of the of formation of surface modified nanoparticles). The reaction was stopped and the surface modified nanoparticles were recovered by centrifugation at 17,000 rpm for 10 min using a high speed refrigerated centrifuge (Avanti J-26XP, Beckman Coulter Inc., Brea, CA, USA). The nanoparticles were thoroughly washed with a mixture of ethanol and deionized water (in ratio of 1:1, three times) using similar centrifugation procedure. The pellets in centrifuge tubes were dispersed with deionized water using sonication and were freeze-dried using a freeze drier. The freeze-dried nanoparticles were crushed and ground using the ceramic mortar and pestle to ensure the production of fine and powdered surface modified SmCNPs (MEEETES-SmCNPs).

Surface modified CNPs with MEEETES functional moieties (MEEETES-CNPs) were also prepared as the control against MEEETES-SmCNPs. MEEETES-CNPs were also produced using a similar method as described. Instead of a combination salts of Ce(NO_3_)_3_·6H_2_O and Sm(NO_3_)_3_·6H_2_O, 5.16 g of pure Ce(NO_3_)_3_·6H_2_O was used as a precursor material and the rest of the steps of the synthesis procedure were kept unchanged.

### 2.2. Characterization

#### 2.2.1. Transmission Electron Microscopy (TEM)

TEM and high resolution TEM (HR-TEM) along with selected area electron diffraction (SAED) measurements of SmCNPs and MEEETES-SmCNPs were carried out using an FEI Titan 80–300 kV (ST) (FEI, Hillsboro, OR, USA) with the field-emission gun operating at 300 kV. Prior to the analysis, the nanoparticles were dispersed by sonication in ethanol, and the small amount of resultant solution was dispersed on a holey carbon film coated on a copper grid. The samples were then mounted on a double-tilt holder and transferred to the microscope for observation of scanning-TEM (S-TEM).

#### 2.2.2. X-Ray Diffraction (XRD)

The powdered X-ray diffraction (XRD) patterns of MEEETES-SmCNPs were collected with a Bruker D8 advance X-ray diffractometer (Bruker Corporation, Billerica, MA, USA) using Cu Kα radiation (λ = 0.154 nm) operated at 40 kV and 40 mA in the Bragg−Brentano geometry using a linear position-sensitive detector with an opening of 2.9°. A nickel filter was used to attenuate contributions from Cu−Kβ radiation. The measurements were performed in a θ–θ mode from 20 to 80 degrees (2θ).

#### 2.2.3. Dynamic Light Scattering (DLS)

DLS measurement of nanoparticles of SmCNP and MEEETES-SmCNP were performed by Zetasizer Nano (Malvern, Worcestershire, UK) at 37 °C in deionized water using a 1 cm path length quartz cuvette. The size distribution and sizes were measured. The hydrodynamic diameter of the nanoparticles (nanoparticle sizes) were expressed as mean ± standard deviation (SD) (*n* = 3).

#### 2.2.4. CP-MAS ^13^C NMR

^13^C nuclear magnetic resonance (NMR) spectra of MEEETES-CNPs and MEEETES-SmCNPs were obtained on a Bruker Avance III spectrometer (Bruker Corporation, Billerica, MA, USA) operating at 14.1 T (600.23 MHz 1H frequency) equipped with Bruker 3.2 mm CP-MAS X/Y/H WB probe head, by polarization transfer from 1H using contact pulse durations of 2 ms at MAS rate of 12 kHz. The temperature for all experiments was kept at 298 K and Bruker TopSpin 3.0 software (TopSpin^®^, Bruker Corporation, Billerica, MA, USA) was sued for data collection and spectral analysis.

## 3. Results and Discussion

The biocompatible surface modification of SmCNPs was carried out in two distinct steps of a single synthetic procedure, which involves the initial step of hydroxylation of SmCNP surface using EG and subsequent reaction of reactive MEEETES with the exposed surface hydroxyl (-OH) groups present over the nanoparticle.

**Figure 1:**
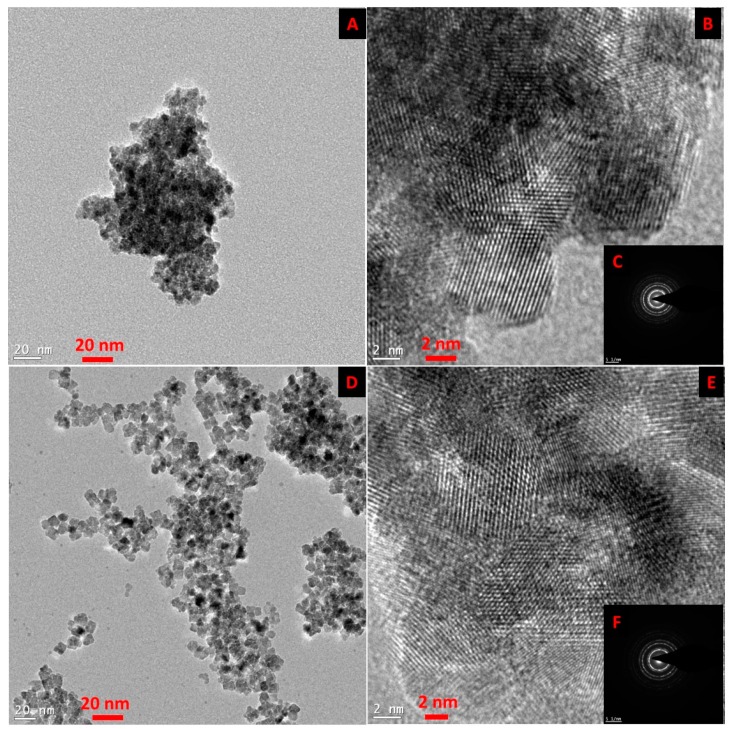
Transmission electron microscopy (TEM) (**A** and **D**), high resolution TEM (HR-TEM) (**B** and **E**), selected area electron diffraction (SAED) (**C** and **F**) of samarium doped cerium oxide nanoparticles (SmCNPs) (**A**, **B**, and **C**), and (6-{2-[2-(2-Methoxy-ethoxy)-ethoxy]-ethoxy}-hexyl)triethoxysilane modified SmCNPs ( MEEETES-SmCNPs) (**D**, **E**, and **F**).

**Figure 2:**
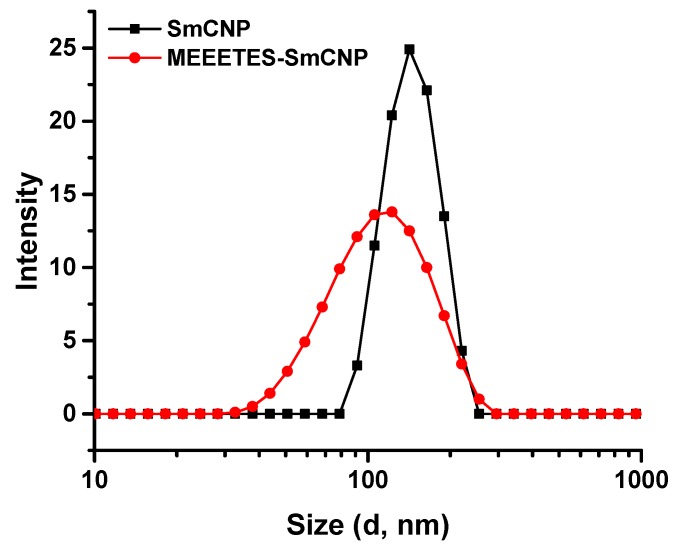
Hydrodynamic size distribution of samarium doped cerium oxide nanoparticles SmCNPs and MEEETES-SmCNPs in aqueous medium.

Figure 1 shows the TEM and HR-TEM images of the SmCNPs and MEEETES-SmCNPs. The microscopic images of the nanoparticles revealed the production of small polyhedral shape nanoparticles nearly 10 nm in size. There were absolutely no significant size differences observed among the individual nanoparticles of SmCNP and MEEETES-SmCNP. The results indicated that the surface modification did not affect the individual nanoparticle size, which means that the MEEETES functional moieties are extremely small. TEM images demonstrated the significant differences in dispersion of SmCNPs at their surface modified and unmodified state. MEEETES-SmCNPs showed more homogeneous dispersion as well as less aggregation than SmCNPs. The information obtained from TEM was further validated using DLS measurement of the nanoparticles in their modified and unmodified conditions (Figure 2).

DLS measures overall nanoparticle size (i.e., the hydrodynamic diameter of the nanoparticles) at their best possible aggregated state [22]. The aggregate size or hydrodynamic diameter (d.nm) was found to have a single significant peak of average particle size (mean Z-Ave) of (236.0 ± 24.1) nm for SmCNPs and (116.4 ± 7.1) nm for MEEETES-SmCNPs. The results indicated that the nanoparticle aggregation was significantly reduced after the surface modification process and had better aqueous dispersibility than SmCNPs as the medium of DLS measurement was essentially aqueous. Since the natural biological environment is essentially aqueous, nanoparticles need to be well dispersible and stable for safe use in biological applications [23]. The improved aqueous dispersibility and higher stability of MEEETES-SmCNPs might be attributed to the surface hydrophilicity in combination with electrostatic and steric repulsion among the nanoparticles after surface modification [24,25].

**Figure 3:**
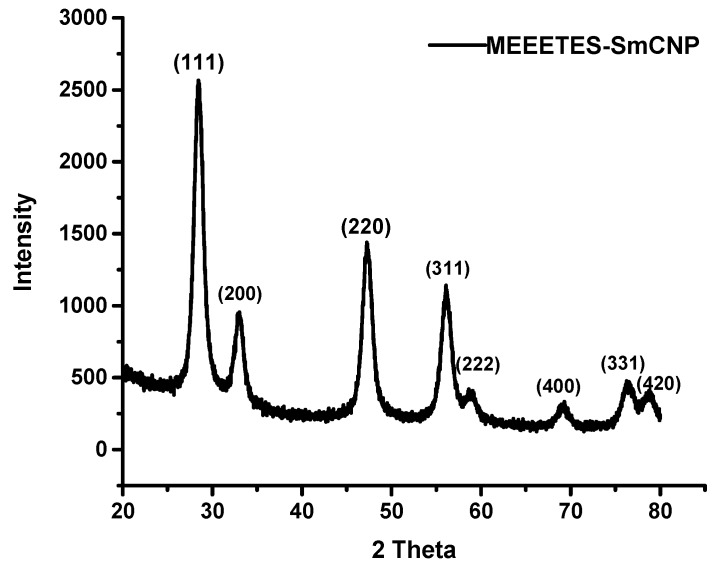
Powdered X-ray diffraction analysis of MEEETES-SmCNPs.

The SAED of both nanoparticles showed ring-like patterns (Figure 1B,F). The images revealed the high crystallinity and ordered structure of the nanoparticles lattice planes. Both SAED and HR-TEM images (Figure 1) demonstrated the lattice fringes could clearly be observed for the nanoparticles at their unmodified and modified state. The powdered XRD pattern of MEEETES-SmCNPs indicated the diffraction peak of the nanoparticles were indexed as nano-CeO_2_ phase (Figure 3) [14]. The characteristic diffraction peaks were marked by their indices (111), (200), (220), (311), (222), (400), (331), and (420) should be well indexed to the face centered cubic (FCC) fluorite structure of available CNPs [14,16]. This indicated that the introduction of samarium did not affect the usual crystal structure of CNPs, as no other additional peaks were observed in the produced diffraction pattern. The results indicated the high crystalline nature of the surface modified redox altered/doped nanoparticles. Moreover, it was noticed that the peaks were broad as an indication of the formation of very fine particles at nanoscale domain.

**Figure 4:**
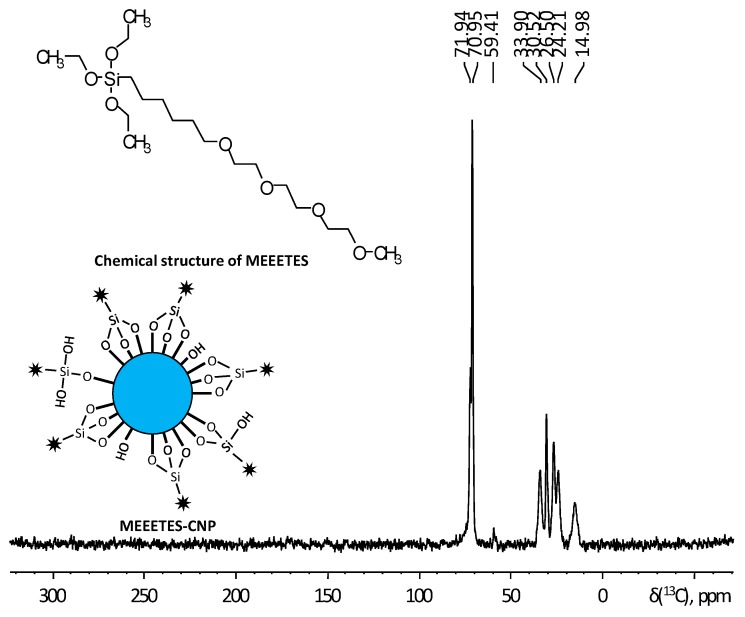
The ^**13**^C CP-MAS spectrum of MEEETES-CNPs with annotated peaks, the chemical structure of MEEETES as a reference, and a schematic drawing showing the possible interaction of MEEETES with CNP in MEEETES-CNPs.

In order to understand the chemical interaction of MEEETES functional moieties with the surface of the redox altered nanoparticles, ^**13**^C CP-MAS solid state NMR was used to study the chemical composition and molecular structure of surface modified nanoparticles. The effect of doping or redox alteration towards the surface modification was studied by comparative analysis among the ^**13**^C NMR spectra obtained from MEEETES-SmCNPs and MEEETES-CNPs. Figure 4 shows the ^**13**^C CP-MAS NMR spectrum of MEETES-CNPs with annotated peaks and a representative chemical structure of MEEETES. NMR spectrum of MEEETES-CNPs showed eight peaks that could be assigned to the different carbon atoms of MEEETES functional moiety. It was expected that ethoxy groups of MEEETES would have given two very strong almost identical NMR signals at 18.2 ppm (**C**H_3_-CH_2_-O-), and at 58.4 ppm (CH_3_-**C**H_2_-O-) [26,27]. The absence of those signals in the spectrum indicated that all alkoxysilanes were bonded to the surface of the nanoparticle via strong chemical interaction, as the nanoparticles were washed thoroughly after the surface modification. All other signals were assigned according to published reports and were in agreement with structure proposed [26,27]. It could be noticed though, that the signal at 14.98 ppm, corresponding to (-Si-**C**H_2_-) group, was distinctly broader than other signals. This might be because not all MEEETES molecules have bonded with all three Si-O-Si bonds, but some only with two or even one, like R-Si(OH)(Si-O-)_2_ or R-Si(OH)_2_(Si-O-). The interaction of MEEETES functional moieties with the surface of the modified CNPs was schematically illustrated in Figure 4.

**Figure 5:**
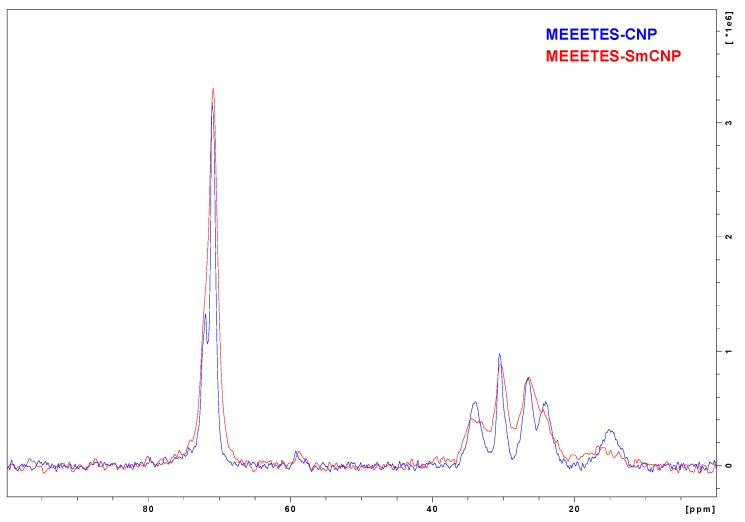
The comparative ^**13**^C CP-MAS NMR spectra of MEEETES-CNPs and MEEETES-SmCNPs.

Figure 5 shows the comparison among the ^**13**^C NMR spectra of MEEETES-SmCNPs and MEEETES-CNPs. It could be noticed that both the spectra of MEEETES-CNPs and MEEETES-SmCNPs were practically identical except for the smaller signal-to-noise ratio in MEEETES-SmCNPs. That could be an indication of a lesser degree of surface modification (or lesser surface area) in MEEETES-SmCNPs compared to MEEETES-CNPs. Since there were no qualitative difference among the spectra of both the surface modified nanoparticles it is of no surprise that the same functionalizing moiety (MEEETES) was used to modify the surface of nanoparticles. However, the main difference was at the signal at around 14 ppm. In MEEETES-SmCNPs, it was found to be broader and shifted to a lower field. That might indicate more functional groups that are connected to nanoparticle surface by only one or two Si-O-Si bonds. However, this does not contradict with the conclusion that all functional MEEETES moieties were bonded to the surface of SmCNPs.

## 4. Conclusions

Cerium oxide nanoparticles are well recognized as a promising antioxidant nanomedicine against several disorders where oxidative stress is a prime concern. Doping and co-doping of CNPs with other trivalent metal ion impurities has been used as an important tool to produce redox altered cerium oxide nanoparticles for multifarious applications in nanobiology and medicine. However, nanobiological application of those doped and co-doped nanoparticles require the particles to be stable with respect to aggregation and effectively dispersible in aqueous media. Our investigation presented a generic approach of preparation and in situ biocompatible surface modification for production of aqueous dispersible redox altered samarium-doped cerium oxide nanoparticles as a model doped nanoparticle of biological relevance. The surface modification using hydrophilic biocompatible (6-{2-[2-(2-methoxy-ethoxy)-ethoxy]-ethoxy}-hexyl) triethoxysilane should also be used as a tool to modify the surface of several other doped and co-doped redox altered cerium oxide nanoparticles for their possible application in nanobiology and medicine.
